# Hypereosinophilia: A Diagnostic Dilemma

**DOI:** 10.4021/jocmr434w

**Published:** 2010-09-20

**Authors:** Habib Ur Rehman

**Affiliations:** University of Saskatchewan, Regina QuAppelle Health Region, Suite 100, 2550, 12th Avenue, Regina, SK, S4P 3X1, Canada. Email:habib31@sasktel.net

## Abstract

A case of hypereosinophilia is presented. The case illustrates the complexity of the diagnostic processes in certain conditions like hypereosinophilia.

**Keywords:**

Hypereosinophilia; Myocarditis; Stroke

## Case Report

A 70-year-old man presented with sudden onset weakness in his right leg of one day’s duration. He denied headaches, chest pains or vomiting. On physical examination the patient was afebrile and appeared well. The pulse was 78/minute and the blood pressure 128/74 mmHg. No lymphadenopathy was found. Examination of the lungs, abdomen, heart, skin, musculoskeletal system was normal. Fundoscopy did not reveal any abnormality. Neurological examination revealed normal strength in upper extremities and left lower extremity. Strength in the proximal muscles of the right leg was 3/5. Reflexes were symmetrical and sensations normal. Cranial nerves were intact.

Past medical history was significant for type 2 diabetes mellitus, recurrent idiopathic angioedema, hyperlipidemia, rheumatoid arthritis and chronic obstructive lung disease. His medication included metformin and glyburide. He travelled to Arizona and Texas every year for 8 years prior to his presentation. He was retired and worked in a steel factory.

Computerized scan (CT) of the brain showed few small ill-defined areas of low density in the superior parietal region on the left side likely representing infarcts due to embolism. Electrocardiogram revealed sinus rhythm and non-specific ST-T wave changes. Short bursts of atrial fibrillation were demonstrated on the rhythm strip. Ejection fraction was 0.55 with normal left ventricular systolic function on transthoracic echocardiogram. Transesophageal echocardiography did not reveal a cardiac source of emboli. Carotid Doppler ultrasound showed minimal amount of plaque within both carotid bulbs. There was no hemodynamically significant stenosis within the common carotid, internal or external carotid arteries bilaterally.

Haemoglobin was 108 g/L, white blood cell 14.7 × 10^9^/L, neutrophils 3.6 × 10^9^/L, lymphocyets 1.1 × 10^9^/L, monocytes 0.5 × 10^9^/L, eosinophils 9.5 × 10^9^/L, basophils 0.0 × 10^9^/L. The platelet count was 218 x 10^9^/L. The levels of urea nitrogen, creatinine, conjugated and total bilirubin, alanine aminotransferase and alkaline phosphatase were normal. Troponin and brain natriuretic peptide (BNP) values were elevated at 2.15 μg/L (< 0.02) and 363.80 ng/L (< 100) respectively. Urinanalysis was normal. Two stool samples were negative for ova or parasites. A review of the outpatient records revealed that eosinophilia was first noticed a year prior to his current presentation.

The levels of immunoglobulins were as follows: IgE 3590 IU/mL (0 - 100), IgG 10.60 g/L (5.52 - 17.24), IgA 2.06 g/L (0.87 - 3.94), and the IgM 1.12 g/L (0.44 - 2.47). Tests for antinuclear antibodies (ANA), antineutrophil cytoplasmic antibodies (ANCA) and antimyeloperoxidase antibodies were negative. Complement C3 level was 1.28 g/L (0.74 - 1.85), complement C4 level was 0.29 g/L (0.16 - 0.44), rheumatoid factor level was < 11 IU/mL (0 - 15), and C1 Inhibitor level was 0.37 g/L (0.21 - 0.39). Immunodiffusion test for aspergillus species was negative. Total leukocyte alkaline phosphatase score was 78 (20 - 146).

CT scan of the chest showed no focal lung lesions. There was quite extensive pleural calcification. There were no pleural effusions and features suggestive of vasculitis. CT scan of the sinuses revealed quite marked mucosal thickening of both maxillary sinuses and extensive mucosal thickening of ethmoidal and frontal sinuses, consistent with a diagnosis of extensive sinusitis. Pulmonary function tests showed normal lung volumes, impaired diffusing capacity down to 58% of the predicted normal and mild air flow obstruction with FEV1 of 3.5L (FEV1/FVC = 64%) without any significant bronchodilator response, diagnosis compatible with chronic obstructive pulmonary disease. Myocardial perfusion scan demonstrated partially reversible perfusion defects involving the septum and distal inferior wall. Additional fixed defect was seen involving the majority of the apex with an associated wall motion abnormality. Cardiac catheterization revealed complete occlusion of the proximal left anterior descending (LAD) artery with rich collateral filling and segmental left ventricular dysfunction but well preserved overall left ventricular contractility.

Endomyocardial biopsy was performed. Sections of endomyocardial biopsy showed fragments of myocardial tissue without significant histopathology. There was no evidence of eosinophilic infiltrate.

Bone marrow aspirate was characterized by diffuse infiltration with eosinophils. Eosinophils including eosinophilic myelocytes formed 75% of the total cell population. Blast count was less than 5%. Erythroid series was normoblastic and myeloid series showed normal maturation. Megakarycytes were present in adequate numbers. Plasma cells formed less than 5%. No ring sideroblasts were seen. Marrow biopsy showed normocellular normoblastic bone marrow. Marked eosinophilia, normoblastic erythroid series and minimal reticulin fibrosis were other features. Bone marrow flow cytometry showed a normal blast population. Cytogenetic analysis of the cultured bone marrow revealed a normal male karyotype. There were no demonstrable clonal karyotypic abnormalities.

## Assessment

The patient presented with monoparesis which raises several possibilities including cerebrovascular accident, peripheral neuropathy, neuromuscular junction disease or a myopathy. Both upper motor neuron weakness and lower motor neuron weakness tend to affect distal muscles in symmetric or asymmetric fashion. Although hypo or hypertonia, and hypo and hyperreflexia would be expected in diseases of the central and peripheral nervous system, these abnormalities are specific but not sensitive and thus when absent are not helpful. Muscle disease should be considered in any patient with symmetric weakness of the proximal muscles of the arms and leg. Asymmetric leg weakness and the absence of dysphagia and neck muscle weakness in our patient make muscle disease unlikely. Disorders of the neuromuscular junction are also unlikely in the absence of ptosis, diplopia and diurnal variation in muscle weakness. Lesions of the ipsilateral spinal cord can also cause monoparesis but associated sensory abnormalities would be expected.

History of type 2 diabetes and hyperlipidemia will make cerebrovascular disease more likely. However, in view of the history of diabetes and frequent travel to Arizona, peripheral mononeuritis due to diabetes or Lyme disease are also possibilities. Rheumatoid arthritis may also be associated with peripheral neuropathies. Lyme disease is unlikely in the absence of other features of Lyme disease like typical rash, fatigue, arthritis and cranial nerve palsies. CT findings are consistent with an embolic ischemic stroke, however no source of the embolism was identified on carotid Doppler and transtesophageal echocardiography.

The most notable blood abnormalities are the presence of striking eosinophilia, raised levels of IgE and troponin levels. Eosinophilia has many causes (Table 1). Troponin rise could be due to myocardial damage due to coronary artery disease or myocarditis. Total serum IgE levels are elevated in a broad array of clinical settings, including infectious, oncologic, inflammatory, allergic, and primary immunodeficiency disorders. Atopy is the most common cause of hypergammaglobulinemia-E worldwide. Infectious causes include filariasis, Schistosomiasis, Toxocariasis, Strongyloidiasis, Trichurasis, Ascariass, Echinococcus, and hookworm infections. These parasitic infections may also cause eosinophilia. Nonparasitic infectious causes of elevated IgE levels include Mycobacterium tuberculosis, Epstein-Barr virus,cytomegalovirus, and human immunodeficiency virus [1]. Gammopathies, Churg-Strauss syndrome (CSS) and several primary immunodeficiency disorders are also associated with elevated IgE levels. Two negative stool samples for parasitic diseases and no history of travel to endemic areas for parasitic infections make parasitic diseases unlikely. Immunoglobulin E-mediated allergy to *Aspergillus* species can cause a brittle form of asthma called allergic bronchopulmonary aspergillosis (ABPA) [2]. ABPA is characterised by asthma, recurrent chest infiltrates on chest radiography, reactive sinusitis, central saccular bronchiectasis, elevated total IgE levels and IgG precipitating antibodies to aspergillus fumigatus. Although our patient had high serum IgE levels, no other features of ABPA were present and immunodiffusion test for aspergillus was negative. Churg-Strauss syndrome, a small vessel vasculitis, is interesting as it could explain the cerebrovascular and myocardial features in our patient and is associated with eosinophilia.

**Table 1:** Causes of Hypereosinophilia and Reasons for Exclusion

Causes of hypereosinophilia	Reasons for exclusion in our patient
Systemic diseases Churg-Strauss syndrome Wegener’s syndrome Polyarteritis nodosa Cholesterol crystal embolism Eosinophilic fasciitis Rheumatoid arthritis	Negative chest X-ray and CT scan Negative ANCA antibodies, negative ANA, negative rheumatoid factor
	
Eosinophilic pneumonias Drug-induced pneumonias Loffler’s syndrome Allergic bronchopulmonary aspergillosis Churg-Strauss syndrome Eosinophilic pneumonia	Normal Chest X-ray and CT results Although high serum IgE levels, no other features of ABPA,negative immunodiffusion test for aspergillus
	
Malignancies and immunodeficiencies Hodgkin lymphoma Non-Hodgkin lymphoma Acute and chronic leukaemia Epithelial cancers Hyper-IgE syndrome Ommen syndrome	Absence of lymphadenopathy clinically and on imaging studies, absence of clonal karyotypic abnormalities. Normocellular normoblastic bone marrow
	
Skin diseases Bullous pemphigoid Cutaneous T-cell lymphoma Systemic mastocytosis Kimura disease Wells disease Eosinophilic pustular folliculitis	No evidence of skin disease
	
Drug-induced eosinophilia Anticonvulsants Non-steroidal anti-inflammatory drugs Antimicrobial agents Sulfa drugs	No history of exposure to drugs known to cause eosinophilia
	
Gastrointestinal diseases Crohn’s disease Eosinophilic gastroenteritis	No clinical features (diarrhea, abdominal pain) suggestive of gastrointestinal disease
	
Infections Helminthic diseases Human immunodeficiency virus (HIV), Human T-lymphocyte virus 1 (HTLV1) Tuberculosis	Negative stool studies, no history of travel to endemic areas No risk factors for HIV or FTLV1 Normal chest X-ray and CT scan

Adopted from reference [2]

History of recurrent angioedema is intriguing. Angioedema is classified into those mediated by bradykinin and those dependent on mast cell degranulation. Hereditary and acquired forms have been described [3]. Angiotensin-converting enzyme (ACE) inhibitor-induced angioedema and angioedema associated with C'1 esterase inhibitor depletion are well recognized. Idiopathic angioedema is a diagnosis of exclusion.

Elevated troponins could be due to either coronary artery disease or myocarditis. Myocardial vasculitis is also a possibility and presents with signs of heart failure. Non-specific ST segment and T wave changes on electrocardiogram and global left ventricular dysfunction with small or moderate sized pericardial effusion on echocardiography are usual findings [4]. It is hard to explain the troponin rise in our patient based on coronary anatomy as the appearance of a totally occluded LAD coronary artery with rich collateral filling probably represented old lesion. In the setting of non-specific ST-T wave changes, slight rise in brain natriuretic peptide (BNP), and coronary angiographic findings, myocarditis would be a more likely cause of the troponin rise. Endomyocardial biopsy in our patient was done 2 weeks after the commencement of high dose steroid therapy. It is possible that the eosinophilic infiltration of the myocardium had regressed by then. The results of normal endomyocardial biopsy therefore do not exclude myocarditis in our patient.

## Differential Diagnosis

The differential diagnosis at this stage would include hypereosinophilic syndrome (HES) and CSS. Eosinophilia and organ damage are two of the overlapping features of HES and CSS. It is not always possible to differentiate the two conditions. Asthma, however, is rare in HES but a cardinal feature of CSS. Combination of sinus disease and marked eosinophilia in the context of a multisystem disorder manifested by cardiomyopathy and central nervous system involvement is suggestive of Churg-Strauss syndrome. The test for ANCA is positive in 50 - 70 percent of cases, and a negative test thus does not rule out the diagnosis [5]. Absence of severe or worsening asthma, no evidence of renal involvement and no pulmonary infiltrates in our patient makes CSS unlikely.

Absence of cytogenetic abnormalities and increase in blast cells and a normal leukocyte alkaline phosphatase score makes myeloproliferative variant (M-HES) unlikely in our patient. Furthermore hepatomegaly and splenomegaly would be expected in M-HES. Flow cytometry did not identify phenotypically aberrant subsets of T-cells, making lymphocytic variant (L-HES) unlikely, although these abnormalities may be discrete and difficult to identify. On the other hand, cardiac complications, evidenced by elevated troponin-I levels in our patient, are more common in M-HES while increased serum IgE levels in our patient may point to a diagnosis of L-HES.

Our patient fits into the category of undefined HES. These patients may present with cardiac, pulmonary or neurologic damage. Thromboembolic disease is common. Endocardial damage and eosinophilic myocarditis is the first manifestation of cardiac disease. Fibrosis of the endocardium results in restrictive cardiomyopathy. A thrombus subsequently forms on the damaged endocardium and may embolise to the brain causing a stroke or transient ischemic attack. A more pronounced eosinophilic count and lack of evidence of vasculitis distinguishes it from CSS [6].

## Management

Patients presenting with potentially life-threatening complications, including cardiac or neurologic involvement, and marked eosinophilia should be treated empirically with high-dose corticosteroids to prevent progression of end organ damage, particularly when infectious causes have been excluded. End organ damage and high eosinophil counts in our patient warranted a course of steroid treatment. Treatment with 60 mg prednisone orally was initiated, with rapid improvement in symptoms and reduction of peripheral eosinophilia to normal range. In patients with aggressive disease unresponsive to high-dose corticosteroids, addition of Imatinib therapy should be considered early. Vincristine should be reserved for patients with rapidly progressive, life-threatening disease unresponsive to high-dose steroids and imatinib therapy [7].

The patient was gradually weaned off prednisone over the next 6 months but required a titration up of the prednisone dose to 40mg daily when the dose was reduced to 5mg daily. This resulted in prompt resolution of eosinophilia. He remains asymptomatic with a normal eosinophil count 1 year later and is being followed up closely.

## Discussion

This case illustrates diagnostic processes involved in a very complex and challenging set of problems. Diagnostic process involves both analytical and non-analytical analysis. It also involves causal reasoning to pin-point the role of factors responsible for the condition of the patient at any time during the course of disease [8]. Non-analytic reasoning is pattern recognition but is usually not useful in complex cases such as presented. Analytic reasoning is the primary strategy when a case is complex or when the clinical findings are unusual [9]. Experience is essential for non-analytic thinking whilst a wide knowledge base and critical and lateral thinking form the bases for analytic thinking.

Eosinophilia, cerebrovascular disease and myocardial damage were the essential "important features" of the case discussed and a differential diagnosis was based on these important positive findings. The wide differential diagnosis was gradually narrowed as more information became available. This was based on both positive findings such as CT results of the brain but also importantly on negative findings such as chest CT results, stool tests for ova and parasites and cytogenetic analysis of bone marrow.

Hypereosinophilic syndromes (HES) are a rare group of heterogeneous disorders characterized by persistent eosinophilia defined as > 1500/μL lasting > 6 months with evidence of eosinophil induced organ dysfunction in which other known causes of eosinophilia has been excluded. Hypereosinophilic Diseases Working Group has established 6 separate categories of hypereosinophilic diseases; myeloproliferative variant (M-HES), lymphocytic variant (L-HES), familial eosinophilia, undefined, overlap, and associated [10]. Diagnosis of HES requires three criteria to be met. First, reactive causes of eosinophilia must be excluded to diagnose HES (Table 1). Second, the criteria of eosinophil-mediated tissue damage must be satisfied. Third, molecular basis of eosinophilia should be determined. This includes peripheral blood molecular genetic analysis for (FIP1L1-PDGFRA) fusion and bone marrow cytogenetic analysis.

Myeloproliferative variant (M-HES) of HES is due to genetic abnormalities leading to clonal expansion of the myeloid linage. Lymphocytic variant (L-HES) is due to overproduction of eosinophilopoietic cytokines by abnormal circulating T-cell subsets [11].

Clinical manifestations of HES may involve heart, skin, lung, nervous system and gastrointestinal tract. Cardiac involvement may manifest as myocarditis, endocardial thrombi or endomyocardial fibrosis causing fatal restrictive cardiomyopathy. Electrocardiogram may show non-specific and diffuse T-wave inversion. Echocardiogram and cardiac magnetic resonance imaging (MRI) are sensitive modalities for the detection of endomyocardial fibrosis and intracardiac thrombi. Endomyocardial biopsy is the gold standard for diagnosis of endomyocardial fibrosis.

Pulmonary symptoms may include dry cough, dyspnoea, and chest discomfort. Radiological findings are non-specific and may include infiltrates or diffuse interstitial pneumonia and fibrosis. Cutaneous manifestations include pruritis, erythematous rash, papules, nodules, eczema-like lesions, and urticaria-like eruptions. Mucosal ulcerations are more common in M-HES, whereas angioedema with elevated IgE levels is suggestive of L-HES variant. Vascular manifestations may be due to small vessel vasculitis or large vessel involvement causing digital necrosis, thrombophlebitis or Budd-Chiari syndrome.

Nervous system manifestations include diffuse encephalopathy, transient ischemic episodes, stroke, and peripheral sensory neuropathies. Abdominal pain, diarrhoea, ascites, pancreatitis and cholangitis may be manifestations of gastrointestinal tract involvement. Hepatomegaly and splenomegaly are common in M-HES.

The familial form of HES is a very rare entity. Autosomal dominant pattern has been described in one kindred [12]. Cases of unexplained eosinophilia that do not meet the other classification criteria may be classed as undefined. This category includes a benign variant with no evidence of organ dysfunction; a complex variant with organ dysfunction but does not meet criteria for myeloproliferative or lymphocytic variant; and a cyclical variant with recurrent angioedema and eosinophilia.

Overlap category includes cases where tissue-specific eosinophilia is restricted to specific organs. This category includes eosinophilic esophagitis, eosinophilic gastroenteritis, eosinophilic pneumonia, and eosinophilia-myalgia syndrome. Associated category is association of eosinophilia with other defined diagnosis such as Churg-Strauss syndrome, systemic mastocytosis, sarcoidosis, human immunodeficiency virus (HIV), or inflammatory bowel disease.

Our patient did not fit into either overlap or associated categories. Gleichs syndrome is characterised by eosinophilia accompanied by recurrent episodes of angioedema, urticaria, pruritis, fever, weight gain, elevated serum IgM, oliguria, and eosinophilia. Our patient did not have these features except episodic angioedema and eosinophilia.

Corticosteroids remain the first line treatment for most patients with HES, with the exception of PDGFRA-associated HES. The most appropriate initial dose and duration of treatment have not been studied but a dose of prednisone ≥40mg has been suggested. Most patients respond to this dose. The dose should than be tapered very slowly to the lowest possible dose whilst monitoring the eosinophil count closely. Evaluation of bone density and adjunctive treatment to prevent bone loss is required in those requiring long-term steroid treatment [9]. Hydoxyurea, interferon-α, imatinib, and vincristine have been used in steroid-refractory cases. Preliminary results with monoclonal antibody therapy have been encouraging and have resulted in prolonged remission rates in the majority of patients [13, 14]. Imatinib mesylate is a tyrosine kinase inhibitor and has activity against the fusion kinase FIP1L1/PDGFRA, responsible for PDGFRA-associated HES. Imatinib mesylate is the first line treatment in patients with myeloproliferative-HES and results in rapid normalization of eosinophil count and clinical features. Allogenic bone marrow transplantation is reserved for patients with FIP1L1/PDGFRA-positive disease who are either resistant or intolerant to imatinib therapy [9].

Hypereosinophilic syndrome includes a variety of disorders without any identifiable cause resulting in a wide variety of clinical manifestations with considerable overlap in clinical and laboratory features. A definitive diagnosis can be difficult because of these overlapping clinical and laboratory features and is further hampered by the fact that these disorders have to be distinguished from other entities associated with eosinophilia and end organ damage. A diagnosis of undefined variant of HES was eventually made by a process of analytic reasoning.
